# A Compend of Pharmacy

**Published:** 1886-06

**Authors:** 


**A Compend of Pharmacy.** By F. E. STEWART, M. D., Ph. G., Quiz Master in Chemistry and Theoretical Pharmacy in the Philadelphia College of Pharmacy; Demonstrator and Lecturer on Pharmacology in the Medico-Chirurgical College, and in the Woman's College, Philadelphia. 12mo.; 196 pages. Philadelphia: P. Blakiston, Son & Co.

This little book is No. 11 of the Quiz-Compend Series. All of the books of this series have been prepared with much care, and are justly held as being far above the average compends. This Compend of Pharmacy is based, by special permission, on Prof. Joseph P. Remington's "Text-Book of Pharmacy," which is now the acknowledged standard authority. The author and publishers have given much time towards making it as complete and concise as possible, and in arranging the types and paragraphs so that it may be easily and quickly referred to, and it will be found a useful guide to the tyro and worker in pharmacy.

